# Digital Mental Health Interventions for Adolescents and Young People: Evaluating Efficacy and Accessibility

**DOI:** 10.7759/cureus.85943

**Published:** 2025-06-13

**Authors:** Dennis Adjei-Boateng, Chinyere L Ikoh Rph.

**Affiliations:** 1 Psychiatry and Behavioral Sciences, Mount Sinai Hospital, John F. Kennedy University School of Medicine, Oceanside, USA; 2 Endocrinology, Diabetes, and Metabolism, John F. Kennedy University School of Medicine, Wilhemstad, CUW

**Keywords:** adolescents, data privacy, informed consent, mental health apps, mental health equity, online counseling, teletherapy, young people

## Abstract

Mental health issues are a significant concern among adolescents and young people. Despite the high prevalence of depression, anxiety, and other mental health issues among these individuals, they do not get adequate health and social services. This gap in conventional care has led to a surge in digital mental health interventions (DMHIs). These approaches include mobile apps, online counseling, virtual reality, and teletherapy. Significant evidence has emerged in favor of DMHIs, with improved patient outcomes in various mental health conditions. However, the long-term effects of these interventions remain to be investigated. Furthermore, there are still significant barriers that limit the widespread application of DMHIs. Barriers to access, including technology availability, digital literacy, socioeconomic factors, and stigma, disproportionately affect marginalized groups. This review summarizes the current evidence regarding the efficacy of different DMHIs in managing various mental health conditions. Furthermore, the review also addresses the role of technological platforms in delivering these interventions and explores ethical concerns such as data privacy, informed consent, and the need for regulatory frameworks.

## Introduction and background

Mental health issues remain a significant concern among young people and adolescents. The World Health Organization (WHO) states that one out of seven young people aged 10-19 years is suffering from a mental illness [1]. Unfortunately, even with such a huge number of cases, young people do not receive adequate measures that would help them overcome mental health issues. Health and social services across the globe frequently overlook their needs, which leaves them vulnerable [2]. This is quite concerning because the incidence of anxiety and depression has been continuously rising in the last few years. The rise is more prominent in girls compared to boys [3,4]. Mental health problems are reported to start from a rather young age. For example, a meta-analysis involving 192 studies disclosed the average age to exhibit any mental problem was as low as 14.5 years of age [5]. When people experience ongoing symptoms, even if they do not quite meet the level for a formal diagnosis, it can be a warning sign of deeper mental health issues down the road. If these early signals are not taken seriously, they can lead to long-term problems that are harder to treat later in life [2]. For young people aged 15 to 29, this is especially important, as suicide is the fourth leading cause of death in this age group. Early action can make a big difference in preventing more serious outcomes [1]. However, despite growing awareness, access to traditional mental health care remains limited for many young individuals. Adolescents have to face a number of barriers that limit them from getting the much-needed support, such as long waiting times, stigma, high costs, etc [6]. Young people today are growing up in an increasingly digital world. While technology has its downsides for young people, like addiction, cyberbullying, depression, and even exploitation, it also holds a lot of potential to improve their well-being [7-9]. Digital solutions can serve as key instruments for curtailing health problems and enhancing life quality. This is more relevant for rural adolescents who otherwise have limited and difficult access to traditional care for mental health conditions [10].

With the proliferation of smartphones, tablets, and internet access, digital mental health interventions (DMHIs) have emerged as a promising solution to these challenges. These interventions include mobile apps, online therapy platforms, and self-guided digital programs. The good part about digital therapies is not just their ease of use but the potential to tap a large market. In addition, young adolescents and young adults are more likely to use technology [11]. Such interventions provide privacy and secrecy, which is particularly useful for people who feel embarrassed or judged about going for a traditional therapy [12]. Digitalization eliminates the geographical and temporal constraints posed by traditional models, as it allows younger individuals to seek assistance at any hour or place [13]. Despite the potential being evident, there is still the question as to whether these digital means are indeed addressing the problems of young people's mental health. It is crucial to assess the effectiveness of digital interventions used for mental health to ensure that they deliver tangible benefits to users. Many of these interventions are based on evidence from cognitive-behavioral therapy (CBT), mindfulness practices, or psychoeducation, yet the effectiveness of translating these strategies into digital formats is still under scrutiny [14]. Some studies have shown promising results, with improvements in mood, anxiety levels, and overall mental well-being [15,16]. However, the outcomes can vary significantly depending on the type of intervention, the level of engagement from the user, and the severity of their mental health condition. It is also important to recognize that while digital tools can provide initial support, they are not a one-size-fits-all solution. The variability in outcomes highlights the need for rigorous research to determine which interventions work best for different individuals and under what circumstances [17,18]. Another important consideration when evaluating DMHIs is their accessibility. While these tools are often touted as being more accessible than traditional mental health care, this assumption does not always hold true in practice [19,20].

The goal of this review is to look at the effectiveness of various forms of digital mental health interventions aimed at adolescents and young adults and how easy it is to use them. By looking at the existing body of research alongside different intervention types, we aim to understand better how effective these tools are and where and how they can be optimized.

## Review

Overview of digital mental health interventions (DMHIs)

There is a notable trend towards e-mental health services in adolescent and young patients. The reason for this can be attributed to many DMHIs that offer care in a comfortable and favorable environment to the younger population. These interventions range from simple mobile apps to more advanced virtual therapy tools.

Types of digital interventions

Different types of DMHIs cater to a range of mental health needs. One of the most prevalent tools is mobile applications. They include tools for self-monitoring emotions, providing therapeutic exercises, or offering interactive modules that guide users [21]. Many apps focus on mood tracking and mindfulness, which help users recognize their emotional patterns and work through distressing thoughts. Examples include 'Headspace' and 'Calm', which are supposed to foster mindfulness and help overcome stress [22]. O'Daffer et al., in their systematic review that included 14 randomized controlled trials (RCTs) on Headspace and one RCT on Calm, reported that Headspace improved depression symptoms in 75% of the cases [22].

Online counseling platforms like BetterHelp or Talkspace allow young people to connect with licensed therapists through messaging or video calls. These services combine the structure of traditional therapy with the flexibility of digital communication [23,24]. Virtual reality (VR) is another emerging tool that creates immersive environments where users can confront anxiety or phobias in a controlled setting [25]. Programs like Limbix are pioneering this space, especially for those suffering from severe anxiety or trauma [26]. Then there are chatbots like Woebot, which offer conversational agents that simulate therapy by guiding users through common therapeutic techniques, providing support during stressful moments, or simply offering a listening ear [27]. Teletherapy, which became especially prevalent during the COVID-19 pandemic, allows individuals to have live therapy sessions via video or phone calls. It has gained traction because it breaks down traditional barriers to therapy, such as distance and time, making it easier for young people in remote areas to access professional help [28]. A meta-analysis reported that teletherapy is comparable to in-person psychotherapy (g=0.04, 95% confidence interval (CI):−0.12 to 0.20) [29]. Social media platforms, though not traditionally designed for mental health interventions, have increasingly become spaces where mental health conversations and support groups thrive. Instagram, TikTok, and Twitter host mental health influencers, advocacy pages, and even professional psychologists who provide bite-sized mental health advice and coping strategies, often in ways that are more digestible for younger users [30].

Theoretical frameworks

DMHIs are not just about making mental health care more accessible; they also integrate evidence-based psychological frameworks to ensure that the support offered is effective. One of the most common frameworks used in digital interventions is CBT [14,31,32]. The cornerstone of a CBT-based approach is to help users understand what thoughts they possess and how those thoughts influence their feelings and actions so as to remove dysfunctional cognition and behaviors. Many applications and web-based programs take users through CBT exercises, which help to change negative thoughts and control symptoms of anxiety or depression [33]. Mindfulness-based therapy is another popular model, particularly in apps like Headspace and Calm. These interventions focus on helping users stay present in the moment, encouraging practices like deep breathing, body scans, and guided meditations [34]. There is evidence suggesting that mindfulness can mitigate stress and enhance the quality of life, which is particularly vital to adolescents who suffer from the overwhelming demands of school, societal expectations, and other normal challenges of development [35]. A study by Gavrilova et al. that investigated the impact of Headspace application on mindfulness mechanisms reported that changes in mechanisms of acceptance-attention were observed from the second week of intervention [36].

Acceptance and commitment therapy (ACT) is also being increasingly integrated into digital tools. This approach helps users accept difficult thoughts and emotions rather than trying to avoid them, promoting mental flexibility. Platforms that use ACT encourage users to identify their core values and take committed actions that align with those values, helping them navigate emotional difficulties without getting stuck in negative cycles [37]. A systematic review of 10 studies reported that ACT in web-based delivery was effective in the management of depression [38]. Exposure therapy, often used to treat phobias or severe anxiety, is another framework being adapted for digital platforms, especially with VR technology [39]. Yoshikawa et al., in their systematic review, reported that digital health-based exposure therapies were effective and comparable to in-person therapies. However, the dropout rate was quite high, which was attributed to the absence of personal connections [40].

Review of clinical outcomes

Numerous studies have evaluated the clinical outcomes of DMHI interventions, particularly in addressing anxiety, depression, attention deficit hyperactivity disorder (ADHD), and eating disorders. Most studies report positive outcomes, particularly in reducing symptoms of anxiety and depression, and improving overall emotional well-being [41-44]. One common finding is that DMHIs offer immediate benefits, especially when individuals are unable or unwilling to access traditional therapy due to stigma, logistical issues, or lack of available services. Furthermore, the evidence shows that the efficacy of DMHIs is comparable to traditional therapy. A meta-analysis by Ebert et al. showed that internet-based CBT significantly reduced depressive symptoms in adolescents, with effect sizes comparable to traditional therapy. The overall effect on anxiety and depression was g=0.72 (95% CI:0.55-0.90) [45]. Another review by Hollis et al. found that DMHIs reduced anxiety symptoms in young people by focusing on stress management and coping strategies, again showing effectiveness similar to face-to-face interventions [46].

Digital platforms that integrate gamification elements, such as interactive scenarios or reward systems, are particularly successful in engaging adolescents [47]. Gamification not only increases engagement but also helps maintain long-term adherence to the intervention, a significant challenge in traditional mental health treatments [48]. A systematic review showed that gamification has a positive effect on adherence [49]. Similarly, a meta-analysis indicated that digital tools using gamification for anxiety and depression showed high user satisfaction and clinically significant reductions in symptoms [50]. However, some other reviews have reported that gamification does not incur any significant advantage to mental health apps [51,52]. For ADHD and related executive function disorders, DMHIs are less prevalent but show promise. Digital platforms for ADHD typically involve cognitive training, behavioral strategies, and medication management tools. These interventions have demonstrated moderate success in improving focus, attention, and behavioral regulation. A study by Bul et al. found that a digital game designed for adolescents with ADHD resulted in improvements in attention span and reduced hyperactivity, though the outcomes were less robust than for anxiety and depression interventions [53].

Eating disorders such as anorexia nervosa and bulimia nervosa present a different set of challenges for digital interventions. Due to the intricate psychological and physical components of eating disorders, DMHIs targeting eating disorders often combine psychoeducation with self-monitoring tools and therapeutic support [54,55]. These interventions have shown mixed results, with some studies indicating that digital platforms can help reduce the frequency of disordered eating behaviors, while others suggest they may not address the underlying psychological drivers as effectively as face-to-face therapy [56]. A review article by Loucas et al. indicated that although DMHIs for eating disorders can help with the provision of care and monitoring of the symptoms, their usefulness in treating the disorders is definitely not at par with conventional treatment due to the complexity of the conditions [57]. Self-help programs on the internet, such as 'Overcoming Bulimia Online', have been effective in minimizing binge episodes, but there is still contention on the effectiveness in comparison with conventional therapy [58,59]. A study by Aardoom et al. found that while users of online programs for eating disorders appreciated the anonymity and convenience, they often required additional support from healthcare professionals to sustain recovery [60]. This suggests that DMHIs for eating disorders may serve as a useful adjunct to traditional therapy rather than a replacement.

Mental health conditions addressed

Anxiety and Depression

Anxiety and depression are the most common mental health issues among adolescents and young people. DMHIs have been extensively studied for their efficacy in treating these conditions. Digital CBT (dCBT) has shown the most consistent results in reducing symptoms of both anxiety and depression. A large number of apps and web-based programs, including e-therapy, are now available, and are based on the conventional framework of CBT. They often mix stress management and coping strategies in the form of modules targeting negative thought patterns. For example, the "SPARX" program, which delivers CBT through a fantasy game format, was found to reduce depressive symptoms in adolescents, with similar outcomes to face-to-face CBT [61]. For anxiety, DMHIs often focus on stress reduction and mindfulness techniques. Platforms like Headspace or Calm offer guided mindfulness exercises that have been found effective in lowering general anxiety levels. A randomized controlled trial by Simonsson et al. found that mindfulness-based digital interventions significantly reduced anxiety symptoms in adolescents over an eight-week period [62]. These interventions are particularly appealing because they can be practiced independently and in short, manageable sessions, making them accessible to busy or reluctant users. These interventions can be particularly game changer in low- and middle-income countries where access to mental health care is limited (Table 1).

**Table 1:** Key findings from studies evaluating digital mental health interventions for depression and anxiety in low- and middle-income countries NRCT - non-randomized controlled trial; PHQ - Patient Health Questionnaire; GHQ - General Health Questionnaire

Authors	Year	Study design	Participants		Male: Female (%)	Intervention	Key Findings
			Intervention	Control			
Ebrahem et al. [63]	2023	NRCT	209	-	-	Telehealth nursing intervention	After the telehealth nursing intervention, the proportion of participants with normal to mild depression increased from 62.6% to 82.8%, and those experiencing moderate to extremely severe anxiety decreased from 55.4% to 21.6%. The intervention also improved stress levels, with 85.2% of participants reporting normal stress post-intervention.
Liu et al. [64]	2023	RCT	49	49	82.7: 17.3	Mobile application	The intervention group showed significantly lower depression levels at T2 compared to the control group (B=-5.76, 95% CI=-9.97 to -1.54, p=0.007). Effect sizes indicated small to moderate reductions in depression favoring the intervention at T1 (Cohen's d=-0.178) and T2 (Cohen's d=-0.535).
Öztoprak et al. [65]	2023	RCT	32	32	0:100	Nurse navigation program-based intervention	The intervention group showed significantly higher mean scores for self-care capability and quality of life compared to the control group (p<0.05). Mean scores for depression and anxiety risk were significantly lower in the intervention group than in the control group (p<0.05).
Hong et al. [66]	2023	Quasi-experimental study	21	23	36.4: 63.6	Nurse-led mHealth intervention	The intervention group demonstrated a significant reduction in depression scores compared to the control group.
Duan et al. [67]	2022	Mixed methods study	382	170	41.7: 58.3	Web-based interventions	The intervention did not significantly impact depression (p=0.60), with small effect sizes ranging from Cohen d=0.07 to 0.31.
Liu et al. [68]	2022	RCT	41	42	44.5: 55.5	AI chatbots	The intention-to-treat analysis indicated a significant decrease in depression symptoms, with PHQ-9 scores showing a marked reduction (F=22.89; p<0.01) for participants in the chatbot test group. Follow-up analysis of completers revealed that the reduction in anxiety symptoms was only significant during the initial four weeks.
Yan et al. [69]	2022	RCT	75	75	-	WeChat-based intervention	At the one-month follow-up, the intervention group showed significantly lower Self-Rating Depression Scale (SDS) scores (48.2 ± 9.5) compared to the control group (58.8 ± 11.2), with a mean difference of 10.6 (95% CI:7.28-13.92, p=0.019), indicating improved outcomes.
Dığın et al. [70]	202	RCT	-	-	-	Text message	On the seventh postoperative day, the SMS group demonstrated notably lower mean scores on the State Anxiety Inventory compared to the control group, with a p-value of 0.001.
Dambi et al. [71]	2022	Pilot study	76	0	34.2: 65.8	Inuka coaching app intervention	There was a significant reduction in depression F2,73=7.67; P < .001 alongside improvements in health-related quality of life p the intervention group.
Garg et al. [72]	2022	Before-after uncontrolled treatment cohort study	126	0	34.9: 65.1	Tele-psychiatry	After the intervention, there was a significant reduction in the GHQ-12 scale.

ADHD

For adolescents with ADHD, digital interventions primarily focus on improving attention, impulse control, and behavioral regulation. One innovative approach is the use of digital games specifically designed to train attention and working memory. The game by "Akili Interactive" is an example of a therapeutic video game that has been tested in clinical trials for ADHD management [73]. An RCT by Kollins et al. showed that adolescents using the game exhibited improved attention scores, though the long-term effects on ADHD symptoms remain to be fully understood [74]. Additionally, digital platforms that help manage medication schedules and track behavioral progress offer a valuable resource for young people with ADHD. These platforms often use reminders, reward systems, and progress tracking to help users maintain consistency in their treatment plans, which is critical for managing ADHD symptoms (Table 2).

**Table 2:** Key findings from studies evaluating digital mental health interventions for ADHD RCT - randomized controlled trial; CAT - Comprehensive Attention Test; CANTAB - Cambridge Neuropsychological Tests Automated Battery; EEG - electroencephalography; ADHD - attention-deficit/hyperactivity disorder; IRS - Impairment Rating Scale; CGI-I - Clinical Global Impressions Scale-Improvement; TOVA - Test of Variables of Attention; API - Attention Performance Index

Authors	Year	Study design	Participants		Male: female (%)	Intervention	Key findings
			Intervention	Control			
Luo et al. [75]	2023	RCT	27	28	87.3	Focus Pocus	After three months of training, the inattentive, hyperactive/impulsive symptoms, as well as inhibition, working memory, learning, and life skills, significantly improved across all three groups of children. Objective EEG activity revealed a consistent increase in relative alpha power in the eyes-open (EO) condition among all three training groups.
Mozaffari et al. [76]	2022	Case-control	20	20	100	RehaCom	RehaCom treatment significantly improved auditory and visual response control in children with ADHD at weeks 0 and 5. There was no observed effect on auditory and visual attention during the same period.
Ha et a. [77]	2022	Pilot study	12	9	66.7	DoBrain app	The intervention led to changes in neural activities on EEG and behavior, but no significant improvements were observed in CAT and CANTAB results. Clinical impression showed improvement in 60% of participants.
Medina et al. [78]	2021	Randomized study	14	14	78.6	KAD_SCL_01	Smart digital cognitive treatments led to improvements in inhibitory control, visuospatial working memory, cognitive flexibility, working memory, and overall executive functioning. Participants also showed enhanced performance in behavioral clinical indexes related to behavior and general executive functioning.
Kollins et al. [79]	2021	Multicenter, open-labelled study	130	76	74.1	AKL-T01	IRS scores significantly improved in both the stimulant group (-0.7, p < 0.001) and non-stimulant group (-0.5, p<0.001) after four weeks of treatment. IRS, ADHD-RS, and CGI-I scores remained stable during the treatment pause and showed further improvement during the second treatment period.
Meyer et al. [80]	2020	RCT	20	20	72	SST training	The treatment group exhibited reduced relative theta power in resting EEG compared to the control group. Parent ratings indicated a trend of improved attention, reflected in decreased inattentive behaviors in the treatment group.
Rajabi et al. [81]	2020	RCT	16	16	100	NF training	Treatment significantly impacted all symptom variables except AD and auditory response control (ARC). Participants in the neurofeedback (NF) condition showed normalization of atypical EEG features, with reduced theta waves and increased sensory motor rhythm (SMR) activity in central zero.
Kollins et al. [74]	2020	RCT	164	173	74.3	AKL-T01	Among patients receiving AKL-T01 (n=180) or control (n=168), mean age was similar at 9.7 and 9.6 years, respectively. Median change from baseline TOVA API was 0.88 (95% CI 0.24-1.49; p=0.0060).
Hahn-Markowitz et al. [82]	2020	RCT	50	49	71	Cog-Fun	Mixed effects ANOVA showed significant Time × Group interaction effects on all parent-reported outcomes, with moderate to large treatment effects maintained at follow-up. Treatment effects were replicated after crossover in the control group and were not moderated by medication.
García-Baos et al. [83]	2019	RCT	14	14	64.3	RECOGNeyes	Eye-tracker group demonstrated significant posttest improvements in impulsivity (p=0.0067), reaction time (p<0.0001), and fixation gaze control (p<0.0001).

Comparison to traditional interventions

When comparing digital mental health interventions to traditional face-to-face therapy, there are several factors to consider, including effectiveness, user engagement, and dropout rates. In terms of clinical outcomes, digital interventions-especially those based on CBT-often show comparable efficacy to traditional therapy for anxiety and depression. A meta-analysis by Andersson and Titov (2014) found that dCBT had similar effect sizes to in-person CBT, particularly when guided by a therapist or mental health professional [84]. Similarly, Wagner et al. reported that internet-based interventions have similar efficacy for depression as face-to-face therapy [85]. However, the degree of therapist involvement often influences outcomes; fully self-guided digital interventions tend to be less effective than those involving some level of human support.

However, dropout rates remain a concern for DMHIs. While digital platforms may initially engage users, keeping them engaged over time can be challenging. The flexibility and anonymity of digital interventions may lead some users to disengage without feeling the same level of accountability they might in face-to-face therapy. Ezawa et al. reported a mean dropout rate of 32% for internet-based CBT for depression [86]. Karyotaki et al., in their meta-analysis, reported that male gender, lower educational level, and co-morbid anxiety symptoms were risk factors for dropout in web-based interventions for depression [87]. Baumel et al. reported even a much lower 15-day and 30-day retention rate of 3.9% for mental health apps [88]. This suggests that while DMHIs offer initial appeal, sustaining long-term engagement may require more structured support, such as regular check-ins with a therapist or built-in accountability features.

One of the main criticisms of DMHIs is the uncertainty surrounding their long-term efficacy. While many studies demonstrate short-term improvements in mental health symptoms, there is less evidence regarding whether these benefits are sustained over time. Long-term follow-up studies are still limited, but the available data suggest that DMHIs may provide lasting benefits when used as part of a broader mental health strategy rather than as standalone treatments. For example, a study by Gold et al. found that adolescents who completed a digital CBT program for depression continued to experience reduced symptoms six months and twelve months post-intervention [89]. However, relapse rates were higher among those who did not have follow-up support from a healthcare professional, highlighting the importance of ongoing care. Similarly, for ADHD, digital interventions that focus on skill-building and behavior modification may offer lasting benefits, but they are most effective when combined with other forms of treatment, such as medication or parental support.

Long-term efficacy is also influenced by the nature of the intervention. Programs that offer ongoing access to mental health resources or tools for self-monitoring are more likely to provide sustained benefits than short-term interventions. However, the rapid pace of technological change means that digital platforms must continually evolve to maintain user engagement and effectiveness. Adolescents and young people may quickly lose interest in an app or platform if it does not keep pace with their changing needs or preferences.

Accessibility and ethical considerations

Barriers to Access and Solutions

The availability of technology is one of the most prominent barriers for adolescents as DMHIs utilize advanced technology, which is not common for everyone. Young people from low socioeconomic backgrounds, as well as those living in rural parts of the world, do not have the required devices nor a smartphone; hence they do not have the ability to take part in DMHIs [90,91]. This problem is worsened in parts of the world where the infrastructure of the internet is poor or data charges are sky-high, which in turn makes it challenging for the youth to use online resources for mental assistance [92]. Digital literacy is another critical barrier. Even if young people do have the required devices, skills and knowledge to use DMHIs may be lacking among them [93]. Furthermore, the costs associated with certain digital health interventions, such as paid apps or subscription services, can be a significant barrier [94]. This creates a divide where only those with financial means can fully benefit from digital health resources. While DMHIs are often promoted as a way to reduce stigma by offering more private and anonymous forms of support, many young people still hesitate to seek help. They might feel ashamed to seek help from friends or parents or even consider the possible repercussions of sharing their struggles with mental health on the internet [95].

To expand the reach of digital mental health resources, various interventions can be employed. The most robust of these is targeting internet penetration, especially in the areas that are most disadvantaged. Building up the supply of high bandwidth internet services in hard-to-reach locations would go a long way in bridging the gaps geographical distances create in the implementation of DMHIs [96-98]. Governments and private sector organizations can collaborate to provide affordable, high-speed internet access to low-income communities. This may require subsidizing internet connectivity for the poor or setting up community initiatives that provide free and/or low-cost connectivity in public places like libraries, schools, and health centers [99,100]. Governments, non-profit organizations, and healthcare providers can work together to subsidize or offer free access to these tools for vulnerable populations. This will allow a greater number of young individuals to get the benefit from these services by diminishing the barriers that are posed by the cost associated with DMHIs [101,102].

Data Privacy and Security

One of the most pressing ethical concerns surrounding DMHIs for adolescents and young people is the protection of personal data [103]. Users of these platforms often share sensitive information about their mental health, thoughts, and emotions, which must be treated with the highest level of confidentiality. Parker et al., in their critical analysis of mental health apps, reported that many apps encouraged users to share their data with an online community. Furthermore, 41% of the apps did not inform users about the collection and use of their information with third parties [104]. Unlike traditional face-to-face therapy, where privacy is governed by well-established laws and practices, DMHIs operate in a digital environment that can be more vulnerable to data breaches and unauthorized access. Many adolescents may not fully understand the implications of sharing their personal data online, making it even more crucial for DMHIs to have clear, transparent privacy policies and robust security measures in place [105]. Moreover, the collection of data through digital platforms often extends beyond what users consciously provide. For example, many apps track user behavior, such as the amount of time spent on the platform or engagement with certain features [88,106]. While this information may assist in enhancing user experience and personalizing services, questions arise regarding the volume of information gathered, its application, as well as the potential users who may utilize such data. Adequate cyber security must be put in place to thwart the possibility of hacking, data being stolen, and information systems being compromised [103].

## Conclusions

Digital mental health interventions represent a critical tool in addressing the growing mental health needs of adolescents and young people. This review has shown that there is a considerable potential of these DMHIs in enhancing the mental health of people, in particular those suffering from anxiety and depression. Their flexibility, accessibility, and their core ability to reach persons in distant, rural, or underserved areas make them integral to contemporary mental health care. Such therapies have the advantage of reducing the stigma, assuring anonymity, and providing rapid relief that is ideally suited to youth who are skeptical about traditional therapy sessions. However, while DMHIs offer promising results, their long-term efficacy on more complex mental health conditions is yet to be further explored. Also, the inequities of access, such as the digital gap, economic considerations, and concerns regarding confidentiality have to be solved to ensure these tools benefit all adolescents, regardless of their background. Initiatives should be made to increase digital literacy and bridge the gap in the availability of required tools for DMHIs.
